# Saliency-Enhanced Content-Based Image Retrieval for Diagnosis Support in Dermatology Consultation: Reader Study

**DOI:** 10.2196/42129

**Published:** 2023-08-24

**Authors:** Mathias Gassner, Javier Barranco Garcia, Stephanie Tanadini-Lang, Fabio Bertoldo, Fabienne Fröhlich, Matthias Guckenberger, Silvia Haueis, Christin Pelzer, Mauricio Reyes, Patrick Schmithausen, Dario Simic, Ramon Staeger, Fabio Verardi, Nicolaus Andratschke, Andreas Adelmann, Ralph P Braun

**Affiliations:** 1 Department of Radio Oncology, University Hospital Zurich Zurich Switzerland; 2 Physics Department Swiss Federal Institute of Technology Zurich Zurich Switzerland; 3 Department of Radio Oncology University Hospital Zurich University of Zurich Zurich Switzerland; 4 Department of Dermatology, University Hospital Zurich Zurich Switzerland; 5 ARTORG Center for Biomedical Engineering Research, University of Bern Bern Switzerland; 6 Department of Radiation Oncology, Inselspital, Bern University Hospital Bern Switzerland; 7 Laboratory for Scientific Computing and Modelling Paul Scherrer Institut Villigen Switzerland; 8 Department of Dermatology, University Hospital Zurich, University of Zurich Zurich Switzerland

**Keywords:** dermatology, deep learning, melanoma, saliency maps, image retrieval, dermoscopy, skin cancer, diagnosis, algorithms, convolutional neural network, dermoscopic images

## Abstract

**Background:**

Previous research studies have demonstrated that medical content image retrieval can play an important role by assisting dermatologists in skin lesion diagnosis. However, current state-of-the-art approaches have not been adopted in routine consultation, partly due to the lack of interpretability limiting trust by clinical users.

**Objective:**

This study developed a new image retrieval architecture for polarized or dermoscopic imaging guided by interpretable saliency maps. This approach provides better feature extraction, leading to better quantitative retrieval performance as well as providing interpretability for an eventual real-world implementation.

**Methods:**

Content-based image retrieval (CBIR) algorithms rely on the comparison of image features embedded by convolutional neural network (CNN) against a labeled data set. Saliency maps are computer vision–interpretable methods that highlight the most relevant regions for the prediction made by a neural network. By introducing a fine-tuning stage that includes saliency maps to guide feature extraction, the accuracy of image retrieval is optimized. We refer to this approach as saliency-enhanced CBIR (SE-CBIR). A reader study was designed at the University Hospital Zurich Dermatology Clinic to evaluate SE-CBIR’s retrieval accuracy as well as the impact of the participant’s confidence on the diagnosis.

**Results:**

SE-CBIR improved the retrieval accuracy by 7% (77% vs 84%) when doing single-lesion retrieval against traditional CBIR. The reader study showed an overall increase in classification accuracy of 22% (62% vs 84%) when the participant is provided with SE-CBIR retrieved images. In addition, the overall confidence in the lesion’s diagnosis increased by 24%. Finally, the use of SE-CBIR as a support tool helped the participants reduce the number of nonmelanoma lesions previously diagnosed as melanoma (overdiagnosis) by 53%.

**Conclusions:**

SE-CBIR presents better retrieval accuracy compared to traditional CBIR CNN-based approaches. Furthermore, we have shown how these support tools can help dermatologists and residents improve diagnosis accuracy and confidence. Additionally, by introducing interpretable methods, we should expect increased acceptance and use of these tools in routine consultation.

## Introduction

### Background

Melanoma is one of the top-5 most common cancers in Switzerland with a standardized incidence ratio per 100,000 inhabitants of 29.8 for men and 24.7 for women [1]. Longitudinal data acquired since 1989 show a linear 100% increase of the standardized incidence ratio for men [1] in the last 30 years. Unfortunately, this is a worldwide trend. According to Arnold et al [2], melanoma incidence and deaths are expected to increase 50% and 68%, respectively, by 2024. However, it is known that if diagnosed on time, skin cancer can be cured with a simple surgical procedure, dramatically increasing the survival rate. The rapid increase in cases every year has not been followed by an increase in the available number of dermatologists. This causes the system to operate inefficiently with increasing waiting times to get access to specialist consultation. To cope with such a situation, a study [3] proposed to provide specific training in skin cancer diagnosis to general practitioners to improve their competence in such cases. The outcome of that study showed a positive impact during a limited period after the initial training, but eventually, the accuracy dropped again after one year. Thus, it is necessary to explore long-term solutions that can support dermatologists to face this pandemic in a reliable way.

The use of deep learning has raised substantial interest in dermatology. Seminal studies such as Esteva et al [4] showed how deep learning algorithms can outperform board-certified dermatologists in certain dermoscopic image triage tasks. More recent studies broadened the scope by developing algorithms for automatic screening and ugly-duckling characterization in wide surface images [5]. Despite such encouraging applications and results, the transition from academic research studies to real-world application is only slowly being addressed. Different surveys showed a favorable position from dermatologists [6] as well as patients [7] with respect to the introduction of artificial intelligence (AI) in routine consultations. However, interpretability [8] and the need of a specialist to supervise the outcome are issues that concern clinical users and patients. When exploring the option of implementing such support tools in real-world consultation, there are 2 main aspects to monitor: on the one hand, diagnosis accuracy and, in the other, diagnosis confidence, since both play a key role. For example, the overdiagnosis of benign lesions as malignant lesions and diagnoses with low confidence cause unnecessary surgeries. A frequent scenario in clinical practice is the surgical removal of atypical-looking benign lesions. Even in the hands of experts, there are 5 benign lesions removed for every 1 melanoma [9], and in the hands of nonexperts, this increases exponentially. Another important fact that needs to be carefully considered is the bias that can be introduced in the user’s decision-making. However, one of the main limitations in the adoption of these tools for nonexpert users comes from the interpretability of such tools, which does not transmit confidence in the predictions even if the demonstrated accuracy is high.

### Use of Image Retrieval in Dermatology

Content-based image retrieval (CBIR) is a powerful tool in medical practice that proposes similar cases to the ones under study, thus mimicking an automatized bibliography search. Dermatology is not an exception, since large data sets of images are traditionally available. Early applications of CBIR in skin lesion categorization relied on the comparison retrieved from text and annotations [10] and progressed to consider colors and shapes [11,12]. However, with the introduction of convolutional neural networks (CNNs), CBIR is now using them as the backbone for feature extraction [13-17]. A pilot study [16] concluded that CBIR was perceived as easy to use and engaging; however, trust still has to be gained for routine use. Furthermore, in another high-impact study [17] where the impact of using CBIR as a support tool was evaluated, one of the conclusions was that users tend to prefer other AI approaches such per-class probability in the long term. The authors suggested exploring other CBIR architectures that might overcome this issue. One possible reason is the lack of interpretability of the retrieval process. To overcome this limitation, recent studies [18-20] evaluated the option of introducing interpretability methods such as saliency maps to guide the CNN feature extraction. These newly proposed architectures were tested using radiography data sets, which present substantial differences with dermoscopic imaging. For this reason, in this study, we proposed an updated version of the algorithm by Silva et al [18] for the specific needs of skin lesion diagnosis, which we refer to as saliency-enhanced CBIR (SE-CBIR).

The benefits of SE-CBIR in routine dermatologic consultations are as follows:

- Improved interpretability: Saliency maps provide a visual representation of the regions of interest considered by the neural network during feature extraction.
- Improved retrieval performance: By guiding feature extraction toward the regions of interests, we reduce the noise and nonrelevant information in the retrieval process.
- More efficient real-world implementation: By improving interpretability, trust by nonexperts should increase, supporting the wider adoption and implementation of such tools.

## Methods

### Data Set Description

For the training, validation, and testing of our algorithm, the HAM10000 data set [21,22] was used. It consists of a total of 10,015 labeled dermoscopic images belonging to 7 different categories of pigmented lesions. The ground truth was determined in more than 50% of images by histopathology, and the other half were either confirmed by follow-up examinations, expert consensus, or in vivo confocal microscopy [22]. Follow-up images of lesions were removed during the retrieval process to maximize the diversity. The HAM10000 data set designations for the 7 different classes were kept. The description for each category is as follows: actinic keratosis, squamous cell carcinoma, and Bowen disease (*akiec*); basal cell carcinoma (*bcc*); benign keratosis (*bkl*; solar lentigo, seborrheic keratosis, and lichen planus-like keratosis); dermatofibroma (*df*); melanoma (*mel*); melanocytic nevi (*nv*); and vascular lesions (*vasc*; angiomas, angiokeratomas, pyogenic granulomas, and hemorrhage). The data set is highly unbalanced, with 67% of the total number of lesions corresponding to melanocytic nevi and 11% to melanoma. To counteract such imbalance, a multiclass focal loss function was used during training.

### Ethical Considerations

In this study, we used a public data set, namely HAM10000 [21,22]. This data set is routinely used by research studies and is available under a Creative Commons Attribution-NonCommercial 4.0 International Public License. Therefore, the use of this data set is not subject to ethics board approval.

### Architecture Description

CBIR algorithms are designed to retrieve images from a data set that are related to the image under study. Their performance is boosted by introducing CNNs for feature extraction and representation. We chose EfficientNetB4 [23] as the backbone for our classifier. The choice was driven by the fact that ensemble models combining different versions of EfficientNet were used by the top teams in International Skin Imaging Collaboration (ISIC) competitions [24]. We profited from transfer learning by initializing the weights using “ImageNet” default values. The EfficientNetB4 top layer was removed and replaced by average pooling, batch normalization, and 2 combinations of a dropout and a dense layer. The output layer features a softmax activation function. We extracted the deep features just after the last convolutional layer and before the classification layers.

**Figure 1:**
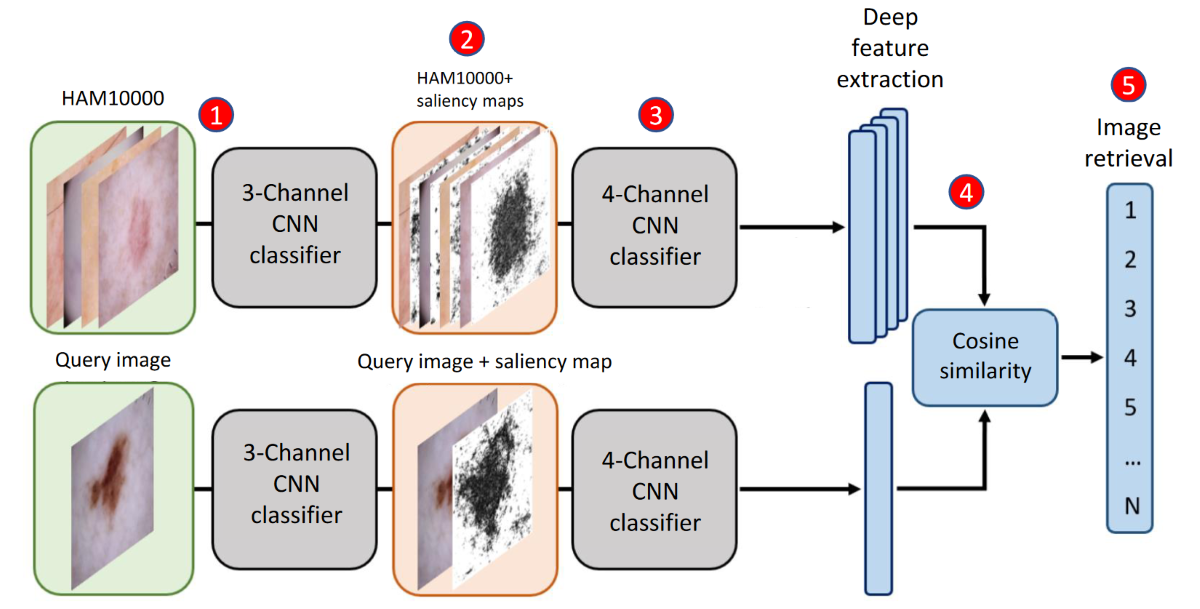
Saliency-enhanced content-based image retrieval (SE-CBIR) scheme. A 7-class classifier is trained in the first stage, from which saliency maps for each image can be extracted. The original classifier is modified by adding an additional channel to input combinations of skin lesion images and their saliency maps for fine-tuning. Finally, the retrieved images are ranked according to the cosine similarity of their deep features. Traditional content-based image retrieval (CBIR) includes only steps 1, 4 and 5, whereas SE-CBIR includes steps 1 to 5. CNN: convolutional neural network.

The traditional CBIR approach, as depicted in Figure 1, includes 3 steps: step 1, a CNN classifier using the HAM10000 data set is trained; step 4, extracted features from the query image are compared to those of each image in the retrieval set; and step 5, images are ranked according to similarity.

In clinical imaging, the relevant information is usually spatially constrained, so new approaches such as that in Silva et al [18] propose to add a fine-tuning stage, guiding the training using saliency maps to enhance the retrieval performance. With the emerging field of explainable AI, a variety of saliency methods have been developed, such as vanilla gradient [25], SmoothGrad [26], and integrated gradients [27]. Saliency methods aim to create a map highlighting the pixels that are relevant for the network’s classification of a particular input image. We chose vanilla gradient for various reasons: it is model agnostic, simple to integrate, interpretable, and consistent. In Silva et al [18], the superiority of latent representations derived from saliency maps over those directly generated from the input x-ray images was demonstrated, where latent representations of saliency maps were used for medical image retrieval purposes and in sample selection for active learning.

Due to the substantial differences between the x-ray images and our dermoscopic data set, we designed a second fine-tune stage that input the original image (3 RGB channels) plus the saliency map computed in the first step. This was done to avoid excessive loss of information included in the original colored dermoscopic images in contrast with the approach in Silva et al [18]. As shown in Figure 1, the traditional CBIR approach is comprised of 3 steps: the training of a skin lesion classifier (step 1), followed by deep feature extraction for every single image (step 4), and finally image retrieval according to similarity score (step 5), whereas SE-CBIR comprises 5 steps, adding the computation of saliency maps (step 2) and the training of a 4-channel classifier (step 3) [28]. It is worth it noting that in step 3, the input and first convolutional layer are expanded to a 4-channel input to input the saliency maps together with the original image.

### Data Augmentation

Standard data augmentation techniques from the *albumentations* libraries [29] were applied during training to avoid overfitting. These transformations include geometric augmentations and noise, distortion, brightness and contrast, and color modifications. The data augmentation is applied on the fly during each epoch. All images were resized and randomly cropped to the expected EfficientNetB4 input resolution (380 × 380). Additionally, coarse dropout to enhance regularization was applied. During the training of SE-CBIR (fine-tuning stage), no data augmentation, other than random flips and rotations, was performed.

### Training

For training, validation, and test purposes, the data set was randomly stratified in an 80:10:10 split, ensuring the same class distribution in all subsets. During training, an adaptative learning rate (α) approach was chosen to allow different α values for the pretrained EfficientnetB4 layers and the added layers, aiming to adapt the parameters of the pretrained layers just slightly or not at all compared to the new layers. A learning rate schedule was set up, with an initial ramp-up during the first epochs that aims to keep the learned features. After the ramp-up, the learning rate decays exponentially. Exponential decay is a widely used learning rate scheduling method to improve convergence. The adaptability of this approach reduces the time required to train neural networks and makes a neural model scalable, as they can adapt to structure and input data at any point in time while being trained.

For step 1, the learning rate was set to a low value (α=5 × 10^–5^) to profit from prelearned features, whereas for the additional layers, the α was set to .01. This approach focuses the training on the classification layers. In this second step, we added an additional channel to the original input size, creating 4D images including the 3 RGB channels plus the 1D saliency maps. An adaptive learning rate approach similar to traditional CBIR was applied. However, the adapted layers were found to perform better at being trained with a 100-times larger (α=1 × 10^–3^) learning rate than that in the first step. The learning rate was kept low at α=1 × 10^–5^ for the pretrained layers to profit again from transfer learning, and the same number of epochs and loss function were used.

The multicategorical focal loss function [30] was chosen for training. This function adds a term to the cross-entropy loss, improving performance in imbalanced data sets such as HAM10000. This is achieved through down weighting, which reduces the influence of easy examples on the loss function, resulting in more attention to hard ones. With a softmax activation function in the last layer, the focal loss ℒ_focal_ for each sample can be derived by:



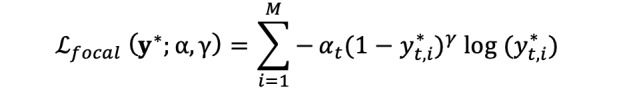



The parameters *α_i_* and *γ* define the weights on this additional term, whereas y*_i_ represents the softmax prediction value of an input.

### Image Retrieval and Ranking Metrics

In CBIR and image classification-based models, high-level image visuals are represented in the form of feature vectors that consists of numerical values. Image retrieval is based on the comparison of features extracted from the images. These vector features are then compared among the different image features to search and rank the “closest” ones. We chose cosine similarity to compare the features’ latent representation due to its adequacy of handling high-dimensional vectors (1792 × 1 in our case). The mathematical representation is as follows:



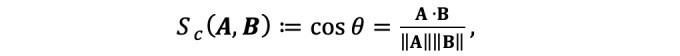



where *A* and *B* are the feature vectors of the query image and each of the images in the labeled data set. The lower the value of *S_c_,* the closer both images are in terms of features extracted.

To evaluate the retrieval performance and compare the traditional CBIR algorithm against SE-CBIR, we used the retrieval precision for *k* retrieved images (*P@k*) or cut-off value metric:



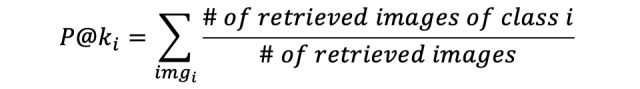



Similarly, for multiclass retrieval, we defined the average precision for *k* retrieved images (*AP@k*):



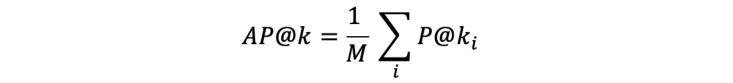



with *M* being the total number of classes.

### Rater Recruitment and Reader Study Design

To evaluate the impact of this type of decision support, a reader study was designed within the University Hospital Zurich Dermatology Clinic. A total of 9 participants were recruited based on their willingness and availability to participate: 1 expert on pigmented skin lesions with more than 25 years of experience and 8 residents from the clinic with 1 to 5 years of experience. However, all of them had reasonable experience with melanoma diagnosis using dermoscopic imaging.

**Figure 2:**
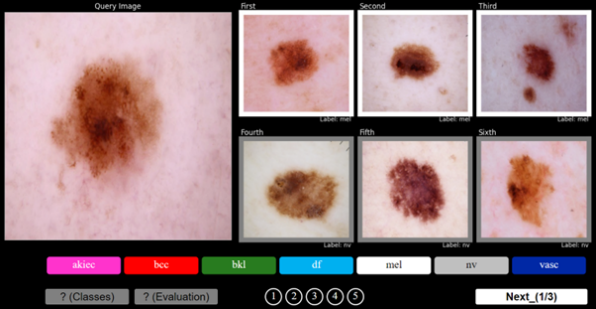
Web interface developed for clinical evaluation. This screenshot corresponds with task 2, where the participants were presented with the query lesion and the 6 closest retrieved ones with their labels (colored edges). The user is asked to specify the confidence for each lesion diagnosis on a scale of 1 to 5. akiec: actinic keratosis, squamous cell carcinoma, and Bowen disease; bcc: basal cell carcinoma; bkl: benign keratosis; df; dermatofibroma; mel: melanoma; nv: melanocytic nevi; vasc: vascular lesions.

To evaluate the impact of the diagnosis support tool, the reader study was divided into 2 tasks. In task 1, volunteers were asked to provide a diagnosis on 100 randomly chosen dermoscopic images extracted from our HAM10000 test set. In parallel, they were also asked to provide their level of confidence in their diagnosis on a 5-point Likert scale [31]. After a break period of 1 day, the participants underwent task 2. They were asked to rediagnose the same set of images while being supported with the 6 “closest” images proposed by the retrieval algorithm that are characterized by their associated ground-truth label, as shown in Figure 2. Note that the saliency maps were not presented to the participants along with the retrievals; however, in a real-world implementation, they would be available. To minimize bias, the single images were rotated by 180° with respect to the original orientation. Additionally, each participant was presented with a different, randomly selected set of images for the evaluation. As in task 1, the volunteers were asked to provide a diagnosis and their confidence level. To facilitate participation, a web survey was created ad hoc for the evaluation process, allowing the users to examine the lesions on high-quality screens and providing flexibility on when and where to perform the evaluation.

## Results

### Saliency Maps

**Figure 3:**
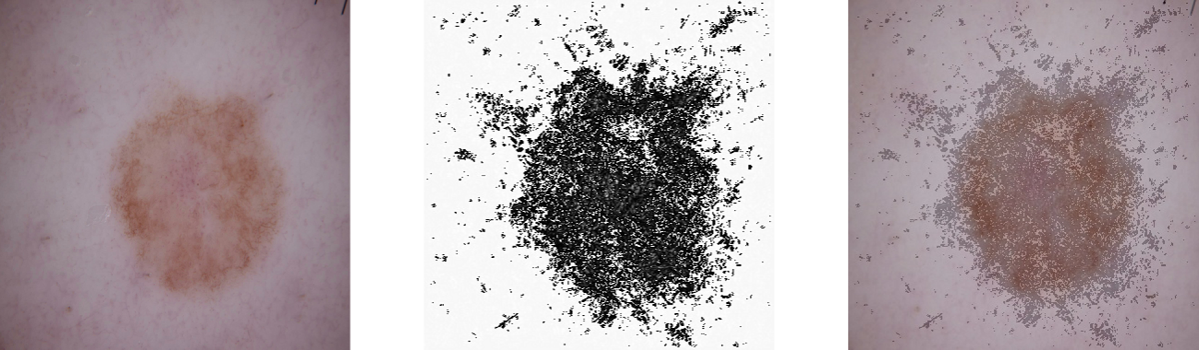
Dermsocopic image (left), extracted saliency map (center), and the overlay of both (right).

An example of a saliency map calculated using the vanilla gradients is shown in Figure 3 (center). Additionally, an overlay of the original image and the saliency map is shown (right) for illustration purposes. To minimize the loss of information during the fine-tuning training step, the original image (3 RBG channels) and the corresponding saliency map (1 channel) are fed as a 4-channel input to the CNN.

### Image Retrieval Results

Retrieval precision comparing the traditional CBIR algorithm versus SE-CBIR is summarized in Table 1. The saliency-enhanced approach improves both the per-class prediction (*P@k*) and the average (*AP@k*) for all values *k* of retrieved images. The improvement in *AP@k* increases with *k*, from 7% (*k*=1) to a maximum of 18% (*k*=9). For a single class, the largest difference was found for class *df* where the prediction accuracy was almost doubled. The only class that did not experience a significant improvement was *akiec*. Both classes *df* and *akiec* were underrepresented with only 1% and 2% of the total retrieval data set. In the case of class *df*, SE-CBIR identified additional features leading to a significant improvement, whereas for class *akiec*, it could be that the limited samples available for class *akiec* presented similar appearances as solar lentigo or seborrheic keratosis and larger statistics are needed. For all other classes, *P@k* was >0.8 for *k*=1.

**Table 1:** Retrieval precision per class for different number of retrieved images comparing the original 3-channel classifier versus the saliency-enhanced one. Precision retrieval was evaluated for k=1, 3, 6, and 9 retrieved images. For each value of k, the average precision was also calculated.

Method and retrieval image (*k*)			Per-class *P@k*^a^								*AP@k* ^b^
		akiec^c^		bcc^d^	bkl^e^	df^f^	mel^g^	nv^h^	vasc^i^		
**SE-CBIR^j^**											
	1	0.59		0.82	0.82	1.00	0.81	0.95	0.93	0.84	
	3	0.57		0.82	0.78	0.97	0.72	0.95	0.88	0.81	
	6	0.56		0.83	0.75	0.98	0.69	0.95	0.89	0.81	
	9	0.56		0.83	0.75	0.96	0.69	0.95	0.90	0.81	
**CBIR^k^**											
	1	0.69		0.75	0.80	0.55	0.67	0.95	1.00	0.77	
	3	0.59		0.67	0.63	0.64	0.54	0.92	0.83	0.69	
	6	0.52		0.66	0.57	0.58	0.49	0.92	0.79	0.65	
	9	0.46		0.65	0.55	0.60	0.47	0.92	0.75	0.63	

^a^P@k: retrieval precision for *k* retrieved images.

^b^AP@k: average precision for *k* retrieved images.

^c^akiec: actinic keratosis, squamous cell carcinoma, and Bowen disease.

^d^bcc: basal cell carcinoma.

^e^bkl: benign keratosis.

^f^df: dermatofibroma.

^g^mel: melanoma.

^h^nv: melanocytic nevi.

^i^vasc: vascular lesions.

^j^SE-CBIR: saliency-enhanced content-based image retrieval.

^k^CBIR: content-based image retrieval.

### Reader Study Outcome

The outcome of the clinical evaluation by the 9 volunteers is summarized in Table 2. Diagnosis accuracy for the SE-CBIR algorithm was computed by majority voting among the *k*=6 retrieved images, which reached 89% on average for all evaluations. In task 1, participants recorded the lowest accuracy at 38%, with an average of 62.2%. The performance was not uniform with σ=12.7 points. As expected, the board-certified dermatologists performed at the same level as the algorithm. In task 2, a significant improvement in diagnosis accuracy was observed for all participants. The average accuracy increased by 22.7 points to 84.9%, which also helped to reduce diagnosis spread, bringing it down to σ=7.1 points. Since each participant was presented with a different image data set, to evaluate the consistency of their selection, the Cohen κ coefficient for each participant for tasks 1 and 2 was calculated, as well as the average among them. The results showed that agreement between raters improved with the support of AI, going from fair agreement (*k_average*=0.35) in task 1 to substantial agreement (*k_average*=0.66) in task 2. In addition, the 2 best-performing participants in task 1 were able to outperform the SE-CBIR majority-voting prediction when provided with retrieved images. It is worth noting that the HAM10000 data set is composed of hand-picked lesions whose diagnosis can be challenging without access to the patient’s context. Regarding the average diagnosis confidence, an improvement from 3.11 to 3.86 was found (+24%). With the support of the retrieved cases, the confidence in the correct diagnosis increased from 3.35 to 4.03. Indeed, most (6/9, 67%) of the participants expressed a confidence level above 4, both in absolute confidence and confidence for correct diagnoses, whereas in task 1, only 1 participant showed that level of confidence. On the other hand, the confidence in incorrectly predicted lesions increased much less (2.72 to 2.90).

Table 2 shows the performance of the retrieval algorithm with an average accuracy of 89.2%. This result is substantially higher than the largest reader study using the ISIC18 data set published by Tschandl et al [17]; however, it used a different test set for the evaluation.

**Figure 4:**
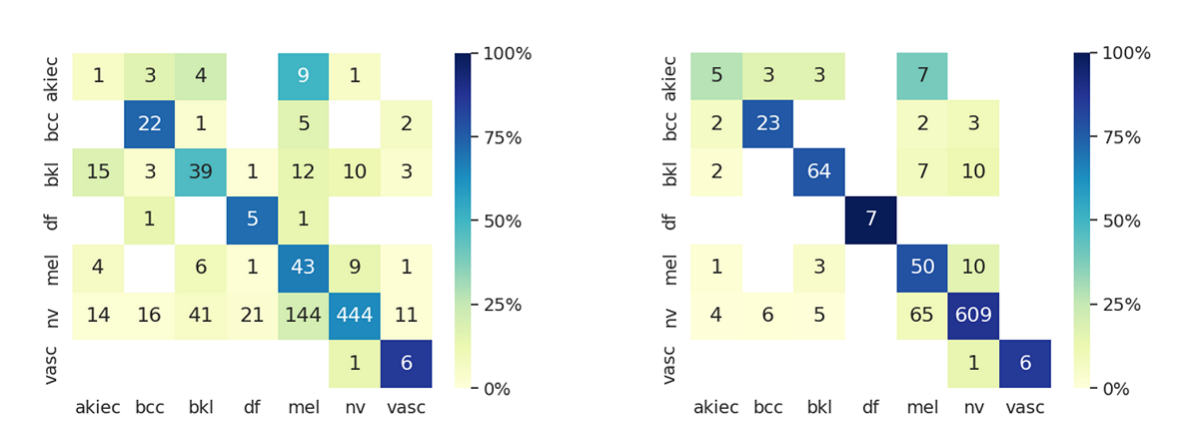
Aggregated confusion matrix for all participants for task 1 (left) and task 2 (right). Overall diagnosis accuracy was improved when using saliency-enhanced content-based image retrieval (SE-CBIR) as a support tool. The melanoma overdiagnosis was reduced by 53% in task 2, mainly driven by changes from initial melanoma diagnosis to nevi. akiec: actinic keratosis, squamous cell carcinoma, and Bowen disease; bcc: basal cell carcinoma; bkl: benign keratosis; df; dermatofibroma; mel: melanoma; nv: melanocytic nevi; vasc: vascular lesions.

A closer look at the predictions per class is shown in Figure 4 with the help of confusion matrices. Each row represents the total number of instances of a given class, whereas each column represents the total number of instances predicted for each class. Figure 4 (right) shows a substantial improvement of per-class accuracy with the help of SE-CBIR–retrieved images.

**Table 2:** Qualitative evaluation results for all participants. Diagnosis accuracy and confidence level on a 5-point Likert scale. Saliency-enhanced content-based image retrieval (SE-CBIR) accuracy was computed by majority voting among 6 retrieved images. The absolute confidence is reported for both tasks, with separate values for correct and incorrect diagnoses.

Participant	SE-CBIR, accuracy (%)	Task 1					Task 2			
		Accuracy (%)	Absolute confidence	Confidence for correct diagnosis	Confidence for incorrect diagnosis	Accuracy (%)		Absolute confidence	Confidence for correct diagnosis	Confidence for incorrect diagnosis
Dermatologist	89	85	3.38	3.48	2.8	92		4.09	4.17	3.13
Resident 1	90	44	2.92	3.45	2.5	84		4	4.12	3.38
Resident 2	89	60	2.43	2.92	1.7	74		2.85	3.31	1.54
Resident 3	92	80	2.51	2.6	2.15	96		3.45	3.51	2
Resident 4	89	62	2.7	2.97	2.26	88		4.41	4.56	3.33
Resident 5	86	66	4.05	4.32	3.53	83		4.46	4.62	3.65
Resident 6	92	65	3.84	3.93	3.66	79		4.06	4.19	3.57
Resident 7	93	60	3.14	3.4	2.75	89		4	4.12	3
Resident 8	88	38	3.04	3.03	3.05	79		3.38	3.56	2.71
Total, mean (SD)	89.2 (2.3)	62.2 (12.7)	3.11 (0.6)	3.35 (0.5)	2.72 (0.6)	84.9 (7.1)		3.86 (0.51)	4.03 (0.5)	2.9 (0.7)

Class *nv* represented almost 80% of the test data set, and the SE-CBIR results show that almost 25% of the diagnoses were reconsidered into the correct class. Similarly, for class *bkl*, incorrect diagnoses were corrected in task 2 improving the accuracy by 17%. For class *mel*, the accuracy improved to 79% for correct melanoma cases. In total, just 20 correct decisions of task 1 were changed to an incorrect diagnosis in task 2, whereas 224 misclassified lesions from task 1 were correctly classified in task 2. This is an important indication that the algorithm does not lead to overconfident misclassifications but rather improves the number of correct diagnoses and their confidence—although we should note that 12 of the incorrectly overturned diagnoses agreed with the algorithm’s majority voting. Those cases probably would require additional patient-context information for a better evaluation. From a skin management point of view, melanoma overdiagnosis is a conservative approach where a negative biopsy might be justified. Regarding melanoma (*mel*) diagnosis, in task 1, a total of 21 cases were underdiagnosed (33% of the 64 total *mel* cases), whereas in task 2, this value decreased to 14 (22%) out of 64 cases. Regarding overdiagnosis in task 1, a total of 171 nonmelanoma cases (where 144 were *nv* cases) were misclassified into *mel*, whereas in task 2, this value decreased to 81 cases (with 65 *nv* cases).

## Discussion

This study presents a novel algorithm for skin lesion diagnosis support based on the use of saliency maps for feature extraction guidance in contrast to state-of-the-art image retrieval (CBIR). We refer to this architecture as SE-CBIR due to the addition of a second fine-tuning stage, combining saliency maps and dermoscopic imaging.

It was shown that SE-CBIR improved retrieval precision in dermoscopic data sets compared to traditional CBIR by 7%. Clinical relevance was assessed by a reader study where the participants improved their overall diagnosis accuracy by +22%, as well their confidence level by +24%. Considering only melanomas, the study demonstrated that SE-CBIR helped to decrease overdiagnosis by 53%.

However, the study has limitations, such as the use of a single data set and the fragility of saliency maps. It is well-known that different methods for saliency map calculation might lead to different results. Another potential limitation identified in the evaluation process is the fact that the participants did not have access to the patient’s context, which penalizes the diagnosis accuracy in certain difficult cases. Future work should include increasing the number of participants, including different target groups such as general practitioners, nurses, or technicians, to evaluate the impact and usefulness of such tools in different scenarios, as well as addressing the imbalance of the classes such as *akiec*, where the current data set does not seem to be representative and is difficult to generalize. In this case, data augmentation techniques will not solve the issue and additional images of such a class should be sought and included.

In conclusion, we have demonstrated the superior quantitative performance of SE-CBIR in comparison to the state-of-the-art CBIR by introducing saliency maps for feature extraction. By introducing interpretable methods, we also expect to improve acceptance by users since they have access to human-understandable information to better comprehend the algorithm’s decision process.

## Abbreviations

AI: artificial intelligence
AP@k: average precision for k retrieved images
CBIR: content-based image retrieval
CNN: convolutional neural network
ISIC: International Skin Imaging Collaboration
P@k: retrieval precision for k retrieved images
SE-CBIR: saliency-enhanced content-based image retrieval

## Data Availability

The origin of the image data set is reported in the data set publication of HAM10000 [22].

The code used in the study is available upon request to the corresponding author for academic purposes.
